# Survival status and predictors of mortality among preterm neonates admitted to neonatal intensive care unit of Addis Ababa public hospitals, Ethiopia, 2021. A prospective cohort study

**DOI:** 10.1186/s12887-022-03176-7

**Published:** 2022-03-23

**Authors:** Dires Birhanu, Bereket Gebremichael, Tewodros Tesfaye, Misrak Tadesse, Fekadeselassie Belege, Yohannes Godie, Markos Wodaje, Eyerusalem Tamiru

**Affiliations:** 1 Dilla University, College of Health Science, Dilla, Ethiopia; 2 Addis Ababa University, College of Health Science, Addis Ababa, Ethiopia; 3 Johns Hopkins University School of Medicine, Baltimore, Maryland, USA; 4 Wollo University, College of Medicine and Health sciences, Dessie, Ethiopia; 5 Gahndi Memorial Hospital, Addis Ababa, Ethiopia; 6 St. Peter Specialized Hospital, Addis Ababa, Ethiopia

**Keywords:** Preterm, Survival, Time to death, Predictors, Addis Ababa

## Abstract

**Background:**

Preterm related complications are the single largest direct cause of neonatal deaths throughout the world, responsible for 35% of the world’s neonatal death (1.1 million deaths/year). In Ethiopia preterm related complications are still the leading cause of neonatal mortality. Identifying the hazard time to death and predictors of mortality play an important role to decrease preterm mortality. Therefore, this study aimed to determine the survival and predictors of mortality among preterm neonates admitted to neonatal intensive care unit of Addis Ababa public hospitals, Ethiopia, 2021.

**Method:**

An institutional based prospective follow up study was conducted among 358 preterm neonates admitted to selected public hospitals of Addis Ababa, Ethiopia from February 12 to May 12, 2021. Systematic random sampling was used to recruit each sample and data was collected prospectively using structured questioner. Epi-data version 4.6 and STATA version 16 was used to data entry and analysis respectively. Kaplan Meier failure curve, Log rank tests were computed. Schoenfeld residual test was used to check overall model fitness. Cox proportional hazards models were fitted to identify independent predictors of preterm mortality.

**Result:**

At the end of this cohort, 125(34.9%) of the neonates died, with incidence rate of 36.4/1000 (CI: 0.031–0.044) person-day with the median time to death of 6 days. Born from antepartum hemorrhage mother (AHR: 3.1, CI; 1.4–6.6), lack of Kangaroo mother care (AHR: 5.8, CI; 2.37–14.33), unable to start feeding with in 24 h of admission (AHR: 6.4, CI: 3.33–12.28), apnea (AHR: 2.4, CI: 1.3–4.7) and dehydration (AHR: 2.33, CI: 1.3–4.3) were the identified predictors of time to death.

**Conclusion and recommendation:**

The first 7 days of admission was the hazard time to death with median time of 6 days. Being born to antepartum hemorrhage mother, lack of Kangaroo mother care, unable to start feeding with 24-h, Apnea and dehydration were the predictors of time to death. Therefore, intervention that focuses on the identified predictors could have a paramount effect to prolong time to death and reduce preterm mortality.

**Supplementary Information:**

The online version contains supplementary material available at 10.1186/s12887-022-03176-7.

## Introduction

Preterm neonates are newborns delivered before 37 completed weeks’ (259th day) of gestation, counting from the first day of the last menstrual period [1–3]. Based on gestational age, preterm neonates can be classified as; extremely preterm (less than 28 weeks), very preterm (28 to 32 weeks), moderate preterm (32 to 33 weeks and 6 days) and late preterm (34 to 36 weeks and 6 days [4].

Globally, 2.4 million neonatal deaths were recorded in 2019 (6700 neonatal deaths per day). One third of all neonatal deaths occur within the first day of life and close to three-quarters occurring within the first week of life [5]. Among the total newborn deaths, three-quarters of it are resulted from complications due to prematurity, intra partum related deaths. Including perinatal asphyxia (PNA) and neonatal infections which can be prevented or treated [6]. Preterm related complications are the single largest direct cause of neonatal deaths throughout the world, responsible for 35% of the world’s neonatal death (1.1 million deaths per year) [7].

In Africa half of the neonatal deaths are from preterm babies caused by only preterm complications and sub-Saharan African countries loses approximately 290,000 neonates in each year due to preterm complication [8]. The highest rates of preterm mortality are in West Africa. nearly 16 per 1000 live births, mainly in Sierra Leone and Liberia [9]. According to the conclusion of an individual participant level meta-analysis study done in east Africa, 52% of newborn deaths are secondary to preterm or neonates who are small for gestational age (SGA) [10]. In Kenya preterm complication (28%) is the second leading causes of neonatal mortality next to intra partum related complications (29%) [11].

Ethiopia is the 4th ranked country in the world which has highest neonatal mortality next to India, Nigeria and Pakistan [12]. Almost 90% of neonatal deaths in the country are due to preterm birth complications (37%), intra partum related complication (28%) and infection (24%) [13]. According to Ethiopian Demography Health Survey (EDHS), neonatal mortality decreased from 39 to 29 from 2005 to 2016, but has increased to 33 in 2019 [14]. Studies in Ethiopia identified several predictors for time to death of preterm mortality such as being male neonate, being born from diabetes mellitus (DM) mother, respiratory distress syndrome (RDS), neonatal sepsis, gestational age less than 28 weeks, low Appearance, Pulse, Grimace, Activity, Respiration (APGAR) score, home delivery, jaundice, hypoglycemia, born from preeclampsia/eclampsia mothers and being extremely very low birth weight [15–17]. However, the studies were conducted in single institution with a retrospective design. Additionally, continuous positive air way pressure (CPAP) types, dehydration, apnea and nurse to patient (neonate) ratio were newly added variables in this study unlike other studies conducted previously.

Furthermore, Ethiopia as a country accepts initiatives to decrease preterm mortality and much is already being done but neonatal death related to preterm complication is still the leading cause of neonatal mortality [18]. Knowing the particular time which is risk for preterm death is vital for policy makers and other stakeholders to intervene accordingly. In addition, predictors of mortality should be identified with regard to time for prevention and management of preterm problems. To do so, further studies should be conducted. Therefore, the purpose of this study was to estimate time to death and predictors of mortality among preterm neonates admitted to selected public hospitals of Addis Ababa.

## Methods

### Study area, design and study period

The study was conducted in Addis Ababa, the capital and largest city of Ethiopia. The city has twelve public hospitals. From these hospitals, the study was conducted in five randomly selected public hospitals of Addis Ababa. These are Tikur Anbessa Specialized Hospital (TASH), Gandihi Memorial Hospital (GMH), Ras-Desta Damtew Memorial Hospital (RDDMH), Yekatit-12 Hospital Medical College (Y12-HMC) and St. Peter Specialized Hospital (SPSH). An institutional based prospective cohort follow up study was conducted from February 12–May 12, 2021.

### Treatment protocol in neonatal intensive care unit

Neonates who need medical care are often put in special units in the hospital called the neonatal intensive care unit. Respiratory support, antibiotic treatment, blood product transfusion, fluid and electrolyte management, phototherapy, thermal stabilization are among the common treatment given in neonatal intensive care unit [19]. CPAP is the most common utilized respiratory support in Ethiopia. There are commonly two types of CPAP which are being applicable in most set-ups. These are Diamediaca and homegrown/homemade types of CPAP. In preterm neonates CPAP plays an important role to prevent collapse of alveoli for this reason it is recommended to put preterm neonates on CPAP as early as delivery. As a general indication neonates with Down score of 4 and above should be kept on CPAP [20].

Early administration of optimal nutrition to preterm birth survivors lowers the risk of adverse health outcomes and improves cognition in adulthood for preterm and low birth weight neonates. In preterm and low birth weight neonates the following are recommended, enteral feeding is safe and may be preferred to parenteral nutrition; early, fast, or continuous enteral feeding yields better outcomes compared to late, slow, or intermittent feeding respectively; routine use of nasogastric tubes is not advisable; preterm infants can be fed while on ventilator or continuous positive airway pressure; routine evaluation of gastric residuals and abdominal girth should be avoided, expressed breast milk is the first choice for feeding preterm infants due to its beneficial effects on cardiovascular, neurological, bone health, and growth outcomes; the second choice is donor pasteurized human milk, optimizing weight gain in preterm infants prevents long-term cardiovascular complications [21].

### Population

All preterm neonates admitted to neonatal intensive care unit (NICU) in selected public hospitals of Addis Ababa are sources of population and all preterm neonates admitted to NICU of selected public hospitals in Addis Ababa in the study period (from Feb 12 to May 12, 2021) were study population.

### Inclusion and exclusion criteria

All alive neonates admitted to NICU by the diagnosis of preterm in selected public hospital with in the study period were included whereas preterm neonates diagnosed with major congenital anomaly (neural tube defect, congenital cardiac disease, gastrointestinal or abdominal wall defect and syndromic babies) were excluded.

### Sample size and sampling procedure

Sample size was calculated by using the STATA version 16 considering the formula N = E/P (E) by taking the hazard ratio and the proportion from previously conducted studies resulted final sample size of 365 by taking the hazard ratio (1.74) of the variables gestational age less than 32 weeks [15] (Additional file 1). This sample size was proportionally distributed to the five randomly selected hospitals by using three-month average base line preterm admission data from patient registration log book. Based on the data GMH had 180, Y12HMC had 118, TASH had 130, RDDMH had 87, and SPSH had 45 average preterm admissions per 3 months. The total population was less than twice of the sample size with k value of 1.53. Therefore, by using systematic sampling technique, from each three consecutive preterm admissions that full fulfill the inclusion criteria, two participants were selected randomly by lottery method until the required sample size was achieved in each hospital. If the recruited sample index mother refuses to participate the next admission was recruited. By using proportional allocation formula 117, 85, 77, 57 and 29 preterm neonates were recruited from TASH, GMH, Y12HMC, RDDMH and SPSH (Additional file 2).

### Study variables

The outcome variable for this study was time to death coded as (death = 1, and censored = 0). Independent variables include:- Maternal socio demographic related predictors; (residence, maternal age, educational status, marital status and occupation), maternal obstetrical and medical predictors; multiple pregnancies, preterm prolonged rupture of membrane (PPROM), mode of delivery, preeclampsia, abruption placenta, antenatal care (ANC) follow up, steroid administration, hypertension, DM, human immune virus/ acquired disease syndrome (HIV/AIDS) and sepsis), Preterm demography related predictors (age at admission, sex, weight, gestational age and weight for gestational age), preterm admission diagnosis and new problem diagnosed in the follow up (APGAR score, diagnosis at admission, new medical between the follow up) and treatment and health service related predictors; (antibiotics, feeding, CPAP, CPAP type, kangaroo mother care (KMC), nurse to patient ratio and feeding).

### Operational definitions/ definition of terms

Appropriate for gestational age (AGA),The percentile of birth weight to gestational age is 10 and 90%) [22]. Died, preterm neonate who died during the follow-up within 28 days after birth and had death summery. Censored, preterm neonates who left the follow up without event (transferred to another institution, left against medical advice, discharged and preterm stays more than 28 days). Event, preterm neonate who died during the follow-up, Extremely low birth weight, neonates born with less than 1000 g of birth weight [22], Extremely preterm: neonates born less than 28 weeks of gestation [4], Feeding: feeding stands for either trophic or full feeding. Follow up time period, Time from recruiting up to either the study subjects died or censored. Large for gestational age (LGA), The percentile of birth weight to gestational age > 90%) [22]. Late preterm, neonates born at 34 to 36 weeks and 6 days of gestation [4]. Low birth weight, neonates born with 1500–2499 g of birth weight [22]. Moderate preterm, neonates born at 32 to 33 weeks and 6 days of gestation [4]. Normal birth weight, neonates with 2500–3999 g of birth weight [22]. Preterm, neonates who are diagnosed as preterm either by last normal mensuration period, by Ballard score or using early ultrasound (20 weeks).SGA, the percentile of birth weight to gestational age is less than10% [22]. Time to death, time at which the neonates died during the follow up. Very low birth weight, neonates born with (1000–1499 g) of birth weight [22]. Very preterm, neonates born from 28 to 32 weeks of gestation [4]. In this study all diagnosis was taken from the treating physician’s diagnoses (who are out of the study team). For example once the physician diagnosed apnea and documented on the neonate’s medical chart, the research team declared that the neonate had apnea until he/she will be improved from the problem. This means that data collectors didn’t diagnose the neonates.

### Data collection tool and procedure

Data collection tool was adapted from different studies with some modification [16, 17, 23]. Study participants were recruited at admission by the data collectors and followed during their stay in the facility, taking note of all significant clinical events until either they become censored or dead. Readmitted neonates were managed according to their previous participation status in the study. In each study site trained nurses collected the data and those data collectors were followed by the supervisors. The principal investigator followed the data collection process. Maternal data were collected by using direct interview and medical chart review. Neonatal data were collected from medical record prospectively until the study participant was died or censored. The maximum time of follow up in the ward was 28 days from birth. To assure the quality of data, the data collection tool was evaluated by neonatologists and research experts, 2 days training was given to the data collectors and supervisors about general research protocols, pretest was done on 5% [20] preterm neonates at Minillik II Memorial hospital for its applicability and appropriateness. Place of delivery and censor type were added to the tool after pretest. Supervisors inspected all the activities of the data collectors and assessed data quality daily. Before the data entry, it was checked for completeness and consistency by the principal investigator.

### Data processing and analysis

Data entry was made by the principal investigator. Preterm death was the event of interest coded as “1” and “0” for censored. Time to death was calculated in days using the time interval between the time of admission and the time of death. Data was entered and cleaned by using EPi Data version 4.6 and transported to STATA version 16 for analysis. Multicollinearity test was done and the variable chorioamnionitis was omitted by the software (STATA) due to multicollinearity with oligo/polyhydramnios.

Log rank test was done to check the existence of significant difference in the survival status among different covariates. Based on this test result marital status and educational status were statistically insignificant (*P*-Value> 0.05). This means that, the Kaplan Meier (KM) failure curve was not statistically different with respect to categories of significant covariates or we have no enough evidence to say that there has difference on failure curve. On the other hand, variables like, birth weight, Gestational age,1st and 5th minute APGAR scores, nurse to patient ratio, apnea, dehydration, necrotizing enterocolitis (NEC), hospital acquired infection (HAI) and preeclampsia/eclampsia has statistically significant result of log-rank test with (*P*-Value < 0.05). This means at 95% confidence, there had difference in hazard time to preterm mortality among the categories (Additional file 3).

Model fitness was tested by using schoenfeld residuals test for proportionality assumption of each covariate and overall model of the stratified cox proportional hazard. Based on this test, the model was fitted with *p*-value of > 0.05 and global test result of 0.146 (Additional file 4). For descriptive statistics frequencies, percentages, rates mean, median and standard deviation were used. KM failure curve was used to show the pattern of death, estimate probability time to death and to compare the failure curves.

Bi-variable cox regression was first fitted and those independent variables having *p*-value ≤0.25 level of significance were included in the multivariable analysis. Cox proportional-hazard regression was fitted at 5% level of significance to determine the net effect of each explanatory variable on outcome variable (HR with its 95% confidence interval and *p*-values were used to measure strength of association and identify statistically significance). *P*-value < 0.05 was considered as statistically significant association. Finally, the results of the study were presented with text, graph and table.

## Result

### Socio-demographic predictors of the study participants with their index mothers

During the study period, there were a total of 593 preterm admission, of those 43 were excluded due to congenital anomaly. A total of 365 neonates with their index mothers were involved initially but 7 preterm neonates were diagnosed with the exclusion criteria after they had been recruited to the study. Response was obtained from 358(98.1%) participants out of these preterm neonates, males were 190 (53.07%) and females were 168 (46.9%). The mean age of the mother was 27.4 ± 4.7SD years. The maximum and minimum maternal ages found in this study were 18 and 40 respectively. In this study 89(24.8%) mothers had no formal learning, 90(25.1%) primary education, 31(8.7%) secondary education 79(22.1%) technical/vocational and 69(19.3%) higher educational level. Most 313(87.4) mothers are living in urban. More than half 221 (61.73%) of the neonates were admitted at the age of less than 24 h and the rest 137 (38.27%) were admitted at the age of 24 and above. The smallest weight and the youngest gestational age recorded in this study were 600 g and 27 weeks respectively (Table 1).

**Table 1:** Socio-demographic characteristics of preterm neonate and their index mothers among those admitted to neonatal intensive care unit of Addis Ababa public hospitals, Ethiopia, 2021

Variables	Categories	Total Number (%)	Status	
			Died (%)	Censored (%)
Marital Status	Single	23 (6.4)	10 (8)	13 (5.5)
	Married	330 (92.2)	112 (89.6)	218 (93.5)
	Divorced	5 (1.4)	3 (2.4)	2 (0.8)
Occupation	Governmental	92 (25.6)	27 (21.6)	65 (27.9)
	Private	87 (24.3)	33 (26.4)	54 (23.2)
	Merchant	32 (8.9)	12 (9.6)	20 (8.5)
	Farmer	4 (1.1)	1 (0.8)	3 (1.3)
	House wife	113 (31.6)	42 (33.6)	71 (30.5)
	Other	30 (8.4)	10 (8)	20 (8.6)
Average household monthly income	Under extreme Poverty	31 (8.7)	15 (12)	16 (6.8)
	Under poverty	51 (14.2)	15 (12)	36 (15.5)
	Above Poverty	276 (77.1)	95((76)	181 (77.7)
Birth Weight	EVBW	15 (41.8)	13 (10.4)	2 (0.9)
	VLBW	99 (27.7)	69 (55.2)	30 (12.8)
	LBW	211 (58.9)	41 (32.2)	170 (73)
	NBW	33 (9.2)	2 (1.6)	31 (13.3)
Gestational age	Extreme Preterm	7 (1.9)	7 (5.6)	0 (0)
	Very Preterm	109 (30.5)	79 (63.2)	30 (12.9)
	Moderate Preterm	59 (16.5)	19 (15.2)	40 (17.2)
	Late preterm	183 (51.1)	20 (16)	163 (69.9)

^a^Other: student, labor work

### Maternal medical, pregnancy and obstetrics related predictors

Almost all 349 (97.5%) index mothers of this study had ANC follow up, of whom around three quarter 238(66.5%) of them had four or more antenatal visit whereas only 39(10.9%) of the mothers have less than four antenatal visits. Around quarters 100 (27.9%) of the mothers had multiple pregnancies, the rest 258(72.1%) had single pregnancy type. From the total index mothers,144(40.2%) had took steroid of them 74(51.4%) got full doses. Almost all 349(97.5%) of the mother had delivered in health institution and only 9(2.5%) gave birth at home. Nearly half of 188(52.5%) the neonates were born via spontaneous vaginal delivery (SVD) and 161(45%) of them were via c-caesarian section (C/S), the rest 9(2.5%) were born instrumentally. Significant majority 316(88.3%) of the mothers had risk for preterm delivery. Around one-third 109(30.4%) of the mothers were preeclamptic and /or eclamptic, 22(6.2%) had oligo/polyhydramnios**,** 124(34.6%) PPROM and 32 (8.9%) had antepartum hemorrhage (APH) (Additional file 5).

### Neonatal admission diagnosis related predictors

Most 313(87.4%) neonates were appropriate for gestational age, only 42 (11.7%) and 3(0.8) of preterm neonates were SGA and LGA respectively. In this study early onset neonatal sepsis (EONS) was found in significant majority of the neonates 309(86.8%) followed by RD 275 (76.8%). More than half 192(53.6%) of the neonates were hypothermic at admission (Table 2).

**Table 2:** Neonatal admission diagnosis related predictors among neonates admitted to neonatal intensive care unit of Addis Ababa public hospitals, Ethiopia, 2021

Variables	Categories	Total (%)	Status	
			Died (%)	Censored (%)
**1st minute APGAR score**	< 3	20 (5.6)	13 (10.6)	7 (3.1)
	3–6	142 (40.2)	59 (47.9)	83 (35.9)
	> = 7	192 (54.2)	51 (41.5)	141 (61)
**5th minute APGAR score**	< 3	3 (0.8)	3 (2.4)	0 (0)
	3–6	59 (16.7)	29 (23.6)	30 (12.9)
	> = 7	292 (82.5)	91 (73.9)	201 (87.1)
**Hypothermia**	Yes	192 (53.6)	87 (69.6)	105 (45.1)
	No	166 (46.4)	38 (30.4)	128 (54.9)
**RDS**	Yes	275 (76.8)	121 (96.8)	154 (66.1)
	No	83 (23.2)	4 (3.2)	79 (33.9)
**EONS**	Yes	309 (86.3)	116 (95.1)	190 (81.5)
	No	49 (13.7)	6 (4.9)	43 (18.5)
**NHB**	Yes	38 (10.6)	2 (1.6)	10 (4.3)
	No	320 (89.4)	123 (98.4)	223 (95.7)
**PNA**	Yes	80 (22.3)	52 (41.6)	28 (12)
	No	278 (77.7)	73 (58.4)	205 (88)

### New medical problem identified during the follow up

Majority 277(77.4%) of the neonate’s had developed new medical problem during the follow up. Of those new medical problems observed during the follow up, 170(47.5%) of the neonates developed hyperbilirubinemia, 98(27.3%) preterm develop HAI, 87(24.3%) develop NEC, 120(33.5%) develop apnea, 136(38%) develop thrombocytopenia and 84(23.5%) were diagnosed for dehydration (DHN) (Additional file 6).

### Treatment related predictors of preterm neonates

More than half of neonates 233(65.1%) were cared by greater than 1 to 2 patient nurse ratio in their hospital stay. Among those preterm babies 232(64.8%) had received CPAP for respiratory support the rest 126(35.2) didn’t get CPAP. Initiation of CPAP in nearly half of the neonates 123(53%) was after admission and only 2(0.8%) of neonates had started CPAP in delivery room before transportation to NICU. The most widely used CPAP type in this study was homemade (homegrown) CPAP. Significant majority 337(94.1%) of preterm neonates had received antibiotics. Among neonates who didn’t start feeding within the first 24 h of admission, 50 (87.7%) were died and only 7(12.3%) survived (Additional file 7).

### Overall survival and mortality of preterm neonates

**Fig. 1:**
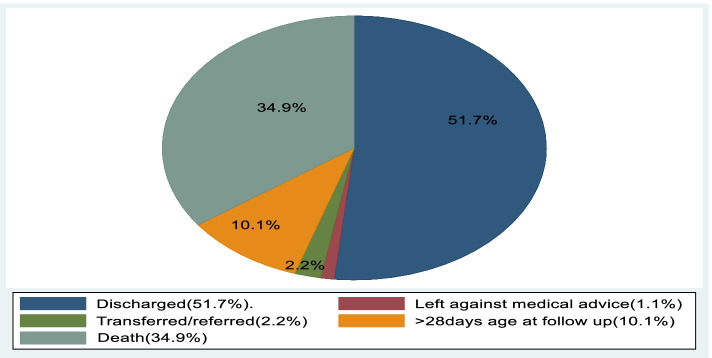
Over all outcomes of preterm neonates admitted to neonatal intensive care unit of Addis Ababa public hospitals, Ethiopia, 2021

In this study a total of 358 preterm neonates were followed for up to 28 days of age starting from admission up to the occurrence of outcome. Among those preterm neonates 125(34.9) of the neonates died with incidence rate of 36.4/1000 neonates-day (Fig. 1).

Most neonates 105 (84%) died in the first 7 days after admission, 8 (6.4%) died before 1 days after admission, 48(38.4%) between one and 3 days from admission, above 3 days up to 7 days of admission 49 (39.2%) and the rest 20(16%) of the neonates died between 7 and 28 days of life (above 7 days of admission). The total incidence rate was 36.4/1000 (CI: 0.031–0.044) neonate - days observation.

The mean, median and standard deviation of time to death of the entire cohort was 9.6, 6 and 8.7 days respectively. The minimum follows up times observed in this cohort was 2 h and the maximum was 28 days. There were no survivors observed in the cohort of less than 28 weeks of gestational age and the survivors of those preterm neonates having less than 1000 g were only two from a total of 15 preterm neonates.

**Fig. 2:**
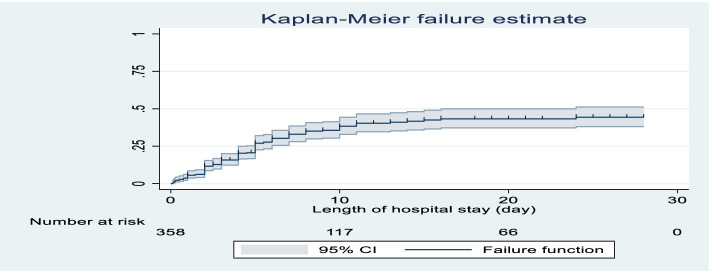
Over all Kaplan-Meier failure estimate of time to death among preterm neonates admitted to neonatal intensive care unit of Addis Ababa public hospitals, Ethiopia, 2021

The cumulative failure of preterm neonates was low in the first day after admission, which increases as follow up time increases up to 28 days of age. At the end of first day of hospital stay the failure was 3.6%, at the end of 3rd day 15.88% which increase to 33.9% at the end of 7 days and 45.5% on 28 day of follow up. As length of hospital stay increases the hazard of time to death will increase (Fig. 2).

### Mortality and comparison of time to death for different categorical variables

In this study neonates having extremely very low birth weight, very low birth weight and low birth weight had higher risk of death than those born with normal birth weight. According to this study, 86.7% of neonates born with birth weight less than 1000 g and 69.7% of the neonates with birth weight of 1000-1499 g were died. The median hazard time to death of extremely very low birth weight and very low were 3 and 5 days respectively which was shorter than time to death of neonates having normal birth weight which was 7 days (Additional file 8).

This study also showed that neonates who developed dehydration during the follow up time had increased risk of death than preterm those didn’t develop dehydration in their stay. The median hazard time to death was 5 days which was shorter than those neonates with no dehydration (7 days) (95%CI: 1.3–4.7) (Additional file 9).

Preterm neonate born to APH mothers had increased risk of death compared to preterm neonate born from mothers who had no APH with the median hazard time to death of 4.5 days and 6 days respectively (95%CI:1.4–6.6) (Additional file 10).

This study also shows that preterm neonates who had developed apnea during the follow-up period were at risk to die with in short period of time than those who didn’t have apnea with the median hospital stay of 4 and 7 days respectively(95%CI:1.3–4.7). The incidence rate was 10.6/100 and the difference was statistically significant with *p*-value of 0.004 (Additional file 11).

Preterm neonates started feeding with in 24 h of admission has favorable survival probability than neonates who had not fed. The median time to death for those hadn’t started feeding and those who had fed were 2 days and 7 days respectively (95%CI: 3.3–12.3). This was statistically significant with *p*-value 0.000 (Additional file 12).

**Fig. 3:**
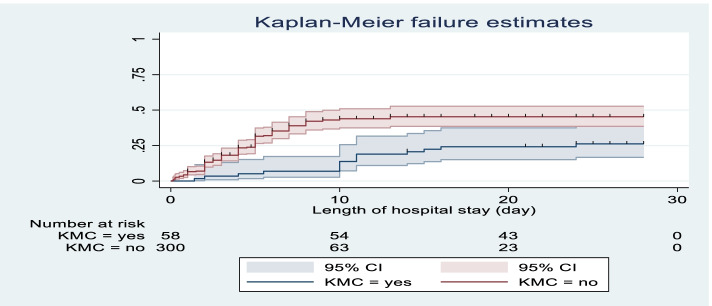
The Kaplan-Meier failure estimates compare time to death of premature neonate with categories of KMC among those admitted to neonatal intensive care unit of Addis Ababa public hospitals, Ethiopia, 2021

In this cohort study preterm neonates who had got KMC had better survival probability than those who hadn’t. The median hazard time to death for neonates without KMC was 5 days which was shorter than those neonates cared with KMC (28 days) with (95%CI: 2.4–14.4). The incidence rate was 11.5/100 to neonates with KMC and 51.3/100 neonates without KMC. This was statistically significant with *p*-value of 0.000 (Fig. 3).

After bi-variable cox regression, variables having *p*-value less than 0.25 were transported to multi-variable cox regression which yields Table 3. Based on this, Apnea (AHR: 2.4), DHN (AHR: 2.33), APH (AHR: 3.1), KMC (AHR: 5.8) and feeding initiation over 24 h (AHR: 6.4) were statistically significant predictors with *p* value< 0.05. This indicates that preterm neonates who developed apnea had 2.4times (CI: 1.3–4.7) shorter time to death compared to those preterm with no apnea during the follow-up. Similarly preterm neonates who developed dehydration during the follow up had 2.33time (CI: 1.3–4.3) shorter time to death than those who didn’t develop dehydration. Preterm neonates born to APH mother had 3.1times (CI: 1.4–6.6) shorter time to death compared to their counter parts. Preterm neonates who hadn’t got care of KMC had 5.8times (CI: 2.37–14.33) shorter time to death than those neonates who had got KMC during the follow up period. Preterm neonates who didn’t start feeding with in 24 h of admission had 6.4times shorten hazard of time to death than their counter parts.

**Table 3:** Multi-variable cox proportional hazard regression result for preterm neonates admitted to neonatal intensive care unit of Addis Ababa public hospitals, Ethiopia, 2021

Variables	Categories	*P*- value	CHR (95% CI)	AHR (95% CI)	Remark
**Apnea**	Yes	0.009	6.4 (4.2,9.66)	2.4 (1.3,4.7)	^a^
	No		1		
**Dehydration**	Yes	0.006	3.5 (2.36,5.0)	2.33 (1.3, 4.3)	^a^
	No		1		
**NEC**	Yes	0.163	2.2 (1.6, 3.2)	.64 (.35, 1.2)	
	No		1		
**HAI**	Yes	0.219	1.6(.92, 1.85)	1.4 (0.8, 2.5)	
	No		1		
**Preeclampsia/eclampsia**	Yes	0.329	2.26 (1.56, 3.25)	1.3(.75 2.29)	
	No		1		
**APH**	Yes	0.004	1.9 (1.2, 3.15)	3.1 (1.4, 6.6)	^a^
	No		1		
**PPROM**	Yes	0.297	1.3(.89, 1.85)	1.3(.78, 2.27)	
	No		1		
**Feeding with in 24 h**	No	0.000	11.4 (7.7,16.8)	6.4 (3.33, 12.28)	^a^
	Yes		1		
**CPAP**	No	0.142	.07(.03, .2)	.39(.11,1.37)	
	Yes		1		
**Hypothermia**	Yes	0.933	2.2 (1.5, 3.2)	1.1(.59, 1.76)	
	No		1		
**RD**	Yes	0.395	9.6 (3.5, 25.9)	1.7(.45, 7.76)	
	No		1		
**EONS**	Yes	0.984	2.8 (1.2, 6.3)	1.(.36, 2.85)	
	No		1		
**PNA**	Yes	0.804	2.9 (2.1, 4.)	1.1(.55, 2.18)	
	No		1		
**Birth Weight**	< 1000	0.105	23.5 (5.3104)	5.13 (0.7, 37.)	
	1001–1499	0.19	13 (3.2, 3.3)	9.5 (1.45, 61.6)	
	1500–2499	0.22	3.1(.75, 12.8)	8.42 (1.4, 52)	
	> = 2500		1		
**KMC**	No	0.000	2.6 (1.5, 4.5)	5.8 (2.37, 14.33)	^a^
	Yes		1		

^a^statistically significant predictors

Variables like marital status, educational status, nurse to patient ratio, gestational age,1st and 5th minute APGAR scores, oligo/polyhydramnios and CPAP type were statistically insignificant in multivariable analysis with *p*-value > 0.05 (Table 3).

## Discussion

This prospective cohort study was aimed to assess the survival and predictors of preterm mortality among preterm neonates admitted to public hospitals of Addis Ababa. Being born to APH mother, apnea and dehydration during the follow-up time, lack of KMC, and inability to started feeding with in 24 h of admission were predictors to time to death. According to this study the overall mortality of preterm neonates admitted to public hospitals of Addis Ababa during the study period was 125(34.9%). This result was similar with the finding of studies done in Jimma university specialized hospital and Bahr Dar university hospital 34.9% [24] and 36.1% [23]. The possible explanation of these similarities could be the study areas were referral hospitals in which different complicated mother can be referred from different parts of the country leads to high flow of preterm and complicated intra-partum complication may leads to preterm death. It can be also explained similarity of NICU setups which haven’t different sophisticated materials that used to save preterm neonates.

However, the finding of this study was higher than the study conducted in China and Iran the 1.9% [25] and 9.1% [26]. The possible explanation for this difference might be due to the difference in neonatal intensive care unit set-ups. Developed countries unlike our NICU set-ups has mechanical ventilators, modern CPAP, parenteral nutrition and infusion pumps which may help to save preterm [27]. The finding of this study was higher than the result done in university of Gondor 28.8% [17], Debre Tabor it was 31.2% [15], Axum university hospital 22.2% [28] and two studies conducted in Addis Ababa 29.7% [16] and 30.7% [29]. The possible explanation for this difference might be due to study design difference. The design of this study was prospective cohort unlike other studies conducted retrospectively. In prospective study missed data due to chart incompleteness is avoided which display all events accordingly. But in retrospective studies death may be masked due to incomplete charts. Another possible reason could be due to study area in which most studies conducted previously done in single institution but this study incorporates many institutions. This may inflate the proportion of death. The difference in exclusion criteria in which different studies excludes preterm neonates born before 28 weeks of gestation might be another possible explanation. In this study preterm neonates born less than 28 weeks of gestation were included unlike other studies in which the proportion of death may be inflated.

The incidence density rate of death in this study was 36.4/1000 neonate-day observation. This finding was lower than the result found in a study conducted in TASH (39.1/1000) neonate-day observation [16]. This difference might be due to the difference in sample size. The study conducted in TASH was conducted in large sample than this study.

This study showed that the highest hazard time was the first 7 days of admission in which majority of the deaths (84%) were encountered. This finding was almost similar with study done in Iran 84.3% [26], Kenya 81.1% [30], in Bair Dar University Hospital 85% [23], in university of Gondor Hospital 85.23% [17]. This might be due to different complication of preterm neonates are happened during pregnancy, at birth and transitional period of neonates from intrauterine to extra uterine or direct complication of pregnancy and abnormal intra-partum process leads to make the neonates to die shortly after birth. Lack of mechanical ventilators may also have contribution to die shortly after birth.

In this study the survival of preterm neonates born at the gestational age of bellow 28,28–32, 32 + 1–33 + 6,34–36 + 6 weeks was 0,38,68 and 89% respectively. This finding was almost similar with the study conducted in Bahir Dar university hospital [23]. This similarity might be due to the similar neonatal intensive care unit service. However, this result is different from a study conducted in Kenya which found that the survival was 29.6% for infants born less than 28 weeks’ gestation, 50% for those born at 28–31 weeks and 75.5% for those born at or above 32 weeks survived [30]. This might be due to neonatal intensive care unit service difference between Kenya and Ethiopia. In Kenya neonatal intensive care unit, surfactant administration and mechanical ventilations which can save preterm neonates from death are available unlike our study areas [31].

In this study, being born from mother having APH was predictors of time to death of preterm mortality which increases the hazard by 3.1times (AHR: 3.1) compared to their counter parts. This was similar with the study conducted in Tanzania [32]. This is supported by the fat that preterm neonates born from APH mother suffer of anemia leads to fetal hypoxia which can increase risk time to death of preterm mortality [33].

Apnea was one of the significant predictor identified in this study which shorten the hazard of time to death by 2.4times (AHR: 2.4). This is supported by the scientific evidence that preterm neonates are vulnerable for intra ventricular hemorrhage (IVH), sepsis and anemia causing for apnea (or it could be apnea of prematurity a diagnosis common in premature babies) [34]. In fact, apnea was diagnosis when babies become critical which may lift out the count in this study.

Preterm neonates developing dehydration during the follow up period were 2.3times (AHR: 2.33) fasten the time to death compared to neonate who didn’t develop dehydration. The possible practical explanation for this reason could be the fact that preterm neonates are vulnerable for excessive water loss due to thin skin and high coverage of water to body mass index. In addition to this, preterm neonates are vulnerable for different medical problems like sepsis and neonatal jaundice which causes dehydration during pathological process and at the time of treatment cascades [35]. Similarly, it could be, due to clinical management gaps such as need for daily weigh, measuring urine output and being able to measure fluid intake - intravenous pumps, proper documentation of intravenous fluid and feeding intake by mouse which are not being done in almost all facilities of this study area. From the above points dehydration would leads to preterm mortality by affecting multi-organ system of the baby.

Unable to start feeding with in the first 24 h of admission 6.4times (AHR: 6.4) fasten the hazard time to death compared to their counter parts. This finding was relatively similar with studies conducting in Uganda [36], Axum University [28] and TASH [16]. The finding is supported by the fact that early initiation of feeding can stimulate gut maturation and breast milk has its own immunoglobulin which can increase the immunity level of preterm neonates, it also stimulate gastro intestinal flora which can used for vitamin-K production [37, 38]. This prevents from the possible bleeding disorder which is common in preterm neonates’ leads to possibly decrease death in relation to time.

Preterm neonates without KMC had 5.8 times shorter time to death compared to neonates who had got KMC. This finding was similar with the study conducted in Uganda Hospital [36], Axum University Hospital [28], and University of Gondor Hospital [17]. The possible expiation might be as preterm neonates are vulnerable for apnea and hypothermia, which can be prevented by KMC. this is supported by the scientific evidence that KMC is a simple intervention to care for preterm newborns by tying the baby to the mother’s front, providing thermal care through continuous skin to skin contact, increased breastfeeding, reduced infections and early recognition of illness [39]. Therefore, lack of KMC leads to this problem which can increase the hazard of death.

### Strength and limitations of the study

This study was conducted prospectively in multicenter institutions by excluding major congenital anomaly. Therefore, it is more representatives and gives qualified data by avoiding the possible missed deaths due to chart incompleteness commonly encountered problem in retrospective studies. Since the study period was only 3 months, it may mask seasonal variability. In this study all preterm neonates were considered as they have equal chance of mortality and survival regardless of their gestational age.

## Conclusion

The incidence rate of preterm mortality was 36.4/1000 neonate-day observation with median time to death of 6 days. Being born to APH mother, lack of KMC, apnea and dehydration during the follow up period, inability to start feeding within 24 h of admission were the identified predictors of time to death. Acting timely on the possible prevention and management of the identified predictors were recommended to prolong time to death and improve survival of preterm neonates.

## Supplementary Information

**Additional file 1.**

**Additional file 2.**

**Additional file 3.**

**Additional file 4.**

**Additional file 5.**

**Additional file 6.**

**Additional file 7.**

**Additional file 8.**

**Additional file 9.**

**Additional file 10.**

**Additional file 11.**

**Additional file 12.**

## Data Availability

The data and other documents used in this study are available from the corresponding author.

## Abbreviations

AGA: Appropriate for gestational age
APGAR: AppearancePulse, Grimace, Activity, Respiration
APH: Ante Partum Hemorrhage
CPAP: Continuous positive air way pressure
DM: Diabetes Mellitus
EDHS: Ethiopian Demographic Health Survey
EONS: Early onset neonatal sepsis
GMH: Gandhi Memorial Hospital
HAI: Hospital Acquired Infection
IVH: Intraventricular hemorrhage
KMC: Kangaroo Mother Care
LGA: Large For gestational Age
NEC: Necrotizing Enterocolitis
NICU: Neonatal Intensive care Unit
PNA: Perinatal Asphyxia
PPROM: preterm prolonged rupture of membrane
RDDMH: Ras-Desta Damtew Memorial Hospital
RDS: Respiratory distress syndrome
SGA: Small for Gestational Age
SPSH: St. Peter Specialized Hospital
TASH: Tikur Anbessa Specialized Hospital
VDRL: Venereal Disease Research Laboratory
WHO: World Health Organization
Y12MCH: Yekatit 12 Medical College Hospital
