# Supplementary material for: Survival status and predictors of mortality among preterm neonates admitted to neonatal intensive care unit of Addis Ababa public hospitals, Ethiopia, 2021. A prospective cohort study
Source: BMC Pediatr. 2022 Mar 23;22:153. doi: 10.1186/s12887-022-03176-7 (PMC8941786; doi:10.1186/s12887-022-03176-7)
Supplement: Supplementary file 1 — Additional file 1. [file 12887_2022_3176_MOESM1_ESM.docx]

**Additional File 1**: Sample size calculation for predictors of preterm mortality among preterm neonates admitted to neonatal intensive care unit of Addis Ababa public hospitals, Ethiopia, 2021.

| Predictors | Hazard Ratio | Adjusting for censoring | Calculated from STATA | Total sample size after 10% non-response rate |
| --- | --- | --- | --- | --- |
| Eclampsia/pre-eclampsia(14) | 1.95 | 0.312 | 251 | 251 |
| **Extremely very low birth weight**(14) | **2.94** |  | **97** | **97** |
| **Gestational age less than 32 weeks**(14) | **1.74** |  | **365** | **365** |
| DM(15) | 2.29 | 0.297 | 172 | 172 |
| Extremely preterm(15) | 2.87 |  | 106 | 106 |
| Low first minute APGAR score (15) | 3.11 |  | 92 | 92 |
