# Supplementary material for: Survival status and predictors of mortality among preterm neonates admitted to neonatal intensive care unit of Addis Ababa public hospitals, Ethiopia, 2021. A prospective cohort study
Source: BMC Pediatr. 2022 Mar 23;22:153. doi: 10.1186/s12887-022-03176-7 (PMC8941786; doi:10.1186/s12887-022-03176-7)
Supplement: Supplementary file 2 — Additional file 2. [file 12887_2022_3176_MOESM2_ESM.docx]

Addis Ababa Public Hospital

Preterm admission in five randomly selected public hospitals (560)

Yekatit 12 MC

118

GMH

180

BLH

130

RDDH

87

SPSH

45

n=117 n=85 n=77 n=57 n=29

**Additional File 2:** Schematic presentation of proportional allocation of to assess survival status and predictors of mortality among preterm neonates admitted to neonatal intensive care unit of Addis Ababa public hospitals, Ethiopia, 2021.
