# Supplementary material for: Survival status and predictors of mortality among preterm neonates admitted to neonatal intensive care unit of Addis Ababa public hospitals, Ethiopia, 2021. A prospective cohort study
Source: BMC Pediatr. 2022 Mar 23;22:153. doi: 10.1186/s12887-022-03176-7 (PMC8941786; doi:10.1186/s12887-022-03176-7)
Supplement: Supplementary file 3 — Additional file 3. [file 12887_2022_3176_MOESM3_ESM.docx]

**Additional File 3:** Median time to death and log-rank test for equality of survivor functions among preterm neonate among neonates admitted to neonatal intensive care unit of Addis Ababa public hospitals, Ethiopia, 2021.

| Variables | Categories | P-value | Median Time to death(day) | Log-rank test (x2) |
| --- | --- | --- | --- | --- |
| **Oliguria/polyhydramnios** | Yes | 0.0006 | 6 | 5.13 |
|  | No |  | - |  |
| **APH** | Yes | 0.0067 | 5 | 6.93 |
|  | No |  | - |  |
| **PPROM** | Yes | 0.0023 | 10 | 1.99 |
|  | No |  | - |  |
| **Feeding within 24 hours** | Yes |  | - |  |
|  | No | 0.000 | 3 | 241.63 |
| **CPAP** | Yes | 0.000 | - |  |
|  | No |  | 4 | 56.01 |
| **Hypothermia** | Yes | 0.000 | 14 | 18.03 |
|  | No |  | - |  |
| **RD** | Yes | 0.000 | 16 | 31.20 |
|  | No |  |  |  |
| **EONS** | Yes | 0.0069 | - | 6.80 |
|  | No |  | - |  |
| **PNA** | Yes | 0.000 | 5 | 39.46 |
|  | No |  |  |  |
| **KMC** | Yes |  | - | 12.44 |
|  | No | 0.0005 | - |  |
| **Types of CPAPA** | Home made | 0.000 | 7 | 61.12 |
|  | Diamedica |  | - |  |
| **Marital Status** | Single |  |  |  |
|  | Divorced | 0.152 | 4 | 3.78 |
|  | Married |  |  |  |
