# Supplementary material for: Survival status and predictors of mortality among preterm neonates admitted to neonatal intensive care unit of Addis Ababa public hospitals, Ethiopia, 2021. A prospective cohort study
Source: BMC Pediatr. 2022 Mar 23;22:153. doi: 10.1186/s12887-022-03176-7 (PMC8941786; doi:10.1186/s12887-022-03176-7)
Supplement: Supplementary file 4 — Additional file 4. [file 12887_2022_3176_MOESM4_ESM.docx]

**Additional File 4:** Schoenfeld's residuals test result among preterm neonates admitted to neonatal intensive care unit of Addis Ababa public hospitals, Ethiopia, 2021.

| Variables | Rho | X2 | Prob>chi2 |
| --- | --- | --- | --- |
| Marital status | 0.16633 | 2.68 | 0.1015 |
| Educational Status | 0.00683 | 0.01 | 0.9410 |
| ANC follow up | 0.04135 | 0.18 | 0.6726 |
| GA | 0.14963 | 3.25 | 0.0715 |
| Weight | 0.00583 | 0.00 | 0.9496 |
| Hypothermia | 0.01712 | 0.04 | 0.8329 |
| RD | 0.00289 | 0.00 | 0.9783 |
| EONS | 0.09502 | 0.72 | 0.3971 |
| PNA | 0.08626 | 0.95 | 0.3304 |
| CPAP | 0.11366 | 1.63 | 0.2012 |
| Types of CPAPA | 0.11329 | 1.62 | 0.2030 |
| Feeding within 24hr | 0.02348 | 0.06 | 0.8039 |
| KMC | 0.10094 | 1.18 | 0.2779 |
| Nurse to neonate Ratio | 0.03679 | 0.18 | 0.6718 |
| PPROM | 0.12807 | 1.99 | 0.1582 |
| APH | 0.10273 | 1.37 | 0.2412 |
| Pre/eclampsia | 0.04183 | 0.25 | 0.6198 |
| Oliguria | 0.01309 | 0.02 | 0.8821 |
| 1^st^ minute APGAR | 0.02534 | 0.07 | 0.7855 |
| 5^th^ minute APGAR | 0.02331 | 0.07 | 0.7897 |
| HAI | 0.17155 | 3.46 | 0.0630 |
| NEC | 0.15180 | 2.92 | 0.0873 |
| Apnea | 0.09022 | 1.08 | 0.2978 |
| Dehydration | 0.12301 | 1.86 | 0.1728 |
| Global test |  |  | **0.1461** |
