# Supplementary material for: Survival status and predictors of mortality among preterm neonates admitted to neonatal intensive care unit of Addis Ababa public hospitals, Ethiopia, 2021. A prospective cohort study
Source: BMC Pediatr. 2022 Mar 23;22:153. doi: 10.1186/s12887-022-03176-7 (PMC8941786; doi:10.1186/s12887-022-03176-7)
Supplement: Supplementary file 5 — Additional file 5. [file 12887_2022_3176_MOESM5_ESM.docx]

**Additional File 5**: Maternal medical, pregnancy and obstetrics related characteristics of index mothers among preterm neonates admitted to neonatal intensive care unit of Addis Ababa public hospitals, Ethiopia, 2021.

| Variables | Categories | Total (%) | Status | |
| --- | --- | --- | --- | --- |
|  |  |  | Died (%) | Censored (%) |
| **VDRL test Result** | Non-reactive | 346(96.7) | 119(95.2) | 227(97.4) |
|  | Reactive | 9(2.5) | 5(4) | 4(1.7) |
|  | Unknown | 3(0.8) | 1(0.8) | 2(0.9) |
| **Steroid administration** | Yes | 144(40.2) | 53(42.4) | 91(39.1) |
|  | No | 214(59.8) | 72(57.6) | 142(60.9) |
| **Dose of Steroid** | One dose | 20(13.9) | 9(16.9) | 11()12.1) |
|  | Two Dose | 27(18.8) | 17(32.1) | 10(10.9) |
|  | Three Dose | 23(15.9) | 7(12.3) | 16(17.7) |
|  | Four doses | 74(51.4) | 20(37.7) | 54(59.3) |
| **PPROM** | Yes | 124(34.6) | 53(44.6) | 71(36.4) |
|  | No | 234(65.4) | 66(55.4)) | 124(63.6) |
| **APH** | Yes | 32 (8.9) | 18(14.4) | 14(6) |
|  | No | 326(91.1) | 107(85.6) | 219(94) |
| **Present of Chronic illness** | Yes | 42(11.7) | 19(15.2) | 23((9.8) |
|  | No | 316(88.3) | 106(84.8) | 210(90.2) |
