# Supplementary material for: Survival status and predictors of mortality among preterm neonates admitted to neonatal intensive care unit of Addis Ababa public hospitals, Ethiopia, 2021. A prospective cohort study
Source: BMC Pediatr. 2022 Mar 23;22:153. doi: 10.1186/s12887-022-03176-7 (PMC8941786; doi:10.1186/s12887-022-03176-7)
Supplement: Supplementary file 6 — Additional file 6. [file 12887_2022_3176_MOESM6_ESM.docx]

**Additional File 6:** New medical problem during the follow-up period among preterm neonates admitted to neonatal intensive care unit of Addis Ababa public hospitals, Ethiopia, 2021.

| Variables | Categories | Total (%) | Status | |
| --- | --- | --- | --- | --- |
|  |  |  | Died (%) | Censored (%) |
| **HAI** | Yes | 98(27.3) | 61(48.8) | 37(15.9) |
|  | No | 260(72.7) | 64(51.2) | 196(84.1) |
| **NEC** | Yes | 87(24.3) | 65(52) | 22(9.4) |
|  | No | 271(75.7) | 60(48) | 211(90.6) |
| **NHB** | Yes | 170(47.5) | 74(59.2) | 96(41.2) |
|  | No | 188(52.5) | 51(40.8) | 137(58.8) |
| **Apnea** | Yes | 120(33.5) | 96(76.8) | 24(10.3) |
|  | No | 238(66.5) | 26(23.8) | 209(89.7) |
| **Thrombocytopenia** | Yes | 136(38) | 71(56.8) | 65(28.9) |
|  | No | 222(62) | 54(43.2) | 168(72.1) |
| **Dehydration** | Yes | 84(23.5) | 65(52) | 19(7.7) |
|  | No | 274(76.5) | 60(48) | 225(92.3) |
