# Supplementary material for: Survival status and predictors of mortality among preterm neonates admitted to neonatal intensive care unit of Addis Ababa public hospitals, Ethiopia, 2021. A prospective cohort study
Source: BMC Pediatr. 2022 Mar 23;22:153. doi: 10.1186/s12887-022-03176-7 (PMC8941786; doi:10.1186/s12887-022-03176-7)
Supplement: Supplementary file 7 — Additional file 7. [file 12887_2022_3176_MOESM7_ESM.docx]

**Additional File 7:** Treatment related predictors among preterm neonates admitted to neonatal intensive care unit of Addis Ababa public hospitals, Ethiopia, 2021.

| Variables |  | Total (%) | Status | |
| --- | --- | --- | --- | --- |
|  |  |  | Died (%) | Censored (%) |
| **Time of CPAP initiation** | Since delivery | 2(0.8) | 0(0) | 2(1.8) |
|  | At admission | 107(46.2) | 31(25.8) | 76(67.8) |
|  | After admission | 123(53) | 89(74.2) | 34(30.4) |
| **Types of CPAP** | Diamedica | 110(47.4) | 48(40) | 62(55.4) |
|  | Homegrown(homemade) | 122(52.6) | 72(60) | 50(44.6) |
| **KMC** | Yes | 58(16.2) | 15(12) | 43(18.5) |
|  | No | 300(83.8) | 110(88) | 190(81.5) |
| **Nurse to patient ratio** | 1:1 | 6(1.7) | 2(1.6) | 4(1.7) |
|  | 1:2 | 119(33.2) | 12(9.6) | 107(45.9) |
|  | >1:2 | 233(65.1) | 111(88.8) | 122(52.4) |
