# Supplementary material for: Survival status and predictors of mortality among preterm neonates admitted to neonatal intensive care unit of Addis Ababa public hospitals, Ethiopia, 2021. A prospective cohort study
Source: BMC Pediatr. 2022 Mar 23;22:153. doi: 10.1186/s12887-022-03176-7 (PMC8941786; doi:10.1186/s12887-022-03176-7)
Supplement: Supplementary file 11 — Additional file 11. [file 12887_2022_3176_MOESM11_ESM.docx]

**Additional File 11:** The Kaplan-Meier failure estimates compare time to death of premature neonate with categories of Apnea among those admitted to neonatal intensive care unit of Addis Ababa public hospitals, Ethiopia, 2021.
