# Supplementary material for: Survival status and predictors of mortality among preterm neonates admitted to neonatal intensive care unit of Addis Ababa public hospitals, Ethiopia, 2021. A prospective cohort study
Source: BMC Pediatr. 2022 Mar 23;22:153. doi: 10.1186/s12887-022-03176-7 (PMC8941786; doi:10.1186/s12887-022-03176-7)
Supplement: Supplementary file 12 — Additional file 12. [file 12887_2022_3176_MOESM12_ESM.docx]

**Additional File 12:** The Kaplan-Meier failure estimates compare time to death of premature neonate with categories of feeding with in 24hour among those admitted to neonatal intensive care unit of Addis Ababa public hospitals, Ethiopia, 2021**.**
